# Nanoparticle Generation in Glowing Wire Generator: Insight into Nucleation Peculiarities

**DOI:** 10.3390/ma14247775

**Published:** 2021-12-16

**Authors:** Elena Fomenko, Igor Altman, Lucija Boskovic, Igor E. Agranovski

**Affiliations:** 1 School of Engineering and Built Environment, Griffith University, Nathan, QLD 4111, Australia; elena.fomenko@griffithuni.edu.au; 2 Combustion Sciences and Propulsion Research Branch, Naval Air Warfare Center Weapons Division, 1 Administration Circle, China Lake, CA 93555, USA; igor.altman2.civ@us.navy.mil; 3 Business and Hospitality Faculty, Torrens University, 90 Bowen Tce, Fortitude Valley, QLD 4006, Australia; lucija.boskovic@Torrens.edu.au

**Keywords:** airborne nanoparticle, glowing wire nanoparticle generator, nanoparticle concentration, controlled nucleation

## Abstract

The paper studies nanoparticle formation in a glowing wire generator (GWG), in which the gas carrier flows around heated metal wire, producing aerosols from a vapor released from the surface. The device has been customized, enabling the use of a double-wire in different orientations in regard to the gas flow. Such alterations provided different effective distances between wires enabling investigation of their mutual influence. Concentration of particles produced in the GWG at different parameters (applied voltage and a gas flow) was carefully measured and analysed. Different regimes of a nanoparticle nucleation were identified that resulted from the applied voltage variation and the gas flow direction. In particular, independent nucleation of nanoparticles on both parts of the wire occurred in the wire plane’s configuration perpendicular to the gas flow, whilst dependent nucleation of nanoparticles was observed at a certain specific set of parameters in the configuration, in which the wire plane was parallel to the gas flow. Two corresponding functions were introduced in order to quantify those nucleation regimes and they tend to zero when either independent or dependent nucleation occur. The peculiarities found ought to be considered when designing the multi-wire GWGs in order to further extend the device’s range for industrial applications.

## 1. Introduction

Different techniques have been suggested for the production of nanoparticles (mainly in the form of aerosols) by atomisation of a gas phase via flame [1], plasma [2], spark discharge [3] and glowing wire generators [4]. Dzimitrowicz et al. [5] investigated the size-controlled synthesis of gold nanoparticles using a glow discharge system equipped with a metallic pin electrode and a flowing liquid electrode at atmospheric pressure. Diameter control of gold nanoparticles (AuNPs) in the region of 2–12 nm was also reported by Shimizu [2] by using an atmospheric-pressure H_2_/Ar regulated plasma jet drive with pulse-modulated ultrahigh frequency, employing Au wire as the source of nanoparticles.

The glowing wire generation method [6] offers precise control over the purity and the size distribution of nanoparticles [7]. These unique features make them an excellent candidate in many industrial applications, including medical [8,9], catalysts [10], electronics [11], gas sensors [12], energy storage [13] and magnetism [14].

The critical properties of these nanoparticles, such as size, shape, purity, oxidation states, and their scale-up manufacturing using a glowing wire generator (GWG), mainly depend on the metal wire type (purity, diameter, electronic stability and resistivity) as well as the employed process parameters such as carrier gas type, flowrate and electric current. The synthesis of tungsten oxide nanoparticles using the glowing-wire technique was reported by Chen and Zhang [4]. They suggested that a steadily and consistently high concentration of nanoparticles could be produced by increasing the electric current to 5.2 A. They also reported that larger-sized particles (count median diameter ~152 nm) could also be generated by adjusting the vapour flowrate and saturated temperature.

Khan et al. [15] investigated the size distribution of metal aerosols in the nanoscale region with a geometric mean ~15 nm using a GWG. They suggested that carrier gas flowrate of 1–1.5 lpm and operating voltage of 6–9 V (equivalent electric power of 30–50 W) could be utilised to produce a steady and continuous number concentration of the metal nanoparticles. Recently, their experimental data has been validated by a theoretical model using wire composition, temperature profile, flow velocity (around the wire), buoyancy effects, as well as the various aerosol dynamics sub-modules including nucleation, coagulation, wall deposition in the formulation [16]. Experimentally averaged and simulated total number concentrations were closely matched for the smaller sized nanoparticles (~10 nm).

Single wire-based GWGs and their crucial process parameters such as gas flowrate, voltage, and the diameter of metal wire have been extensively used to produce metal aerosols. Up to recently, most of the investigations were focused on synthesising smaller size range nanoparticles (less than 30 nm) using this technology. At the same time, larger-sized particles (~100 nm or more) could also have a potential use in many different applications, such as the gas sensor field, photochromic, electrochromic and catalyst. The natural way to enlarge nanoparticles produced in GWGs is to increase the particle formation path in a generation zone [17,18]. In a GWG, it can be achieved by adding additional wires. However, nanoparticle formation in multi-wire GWGs has peculiarities that have not been thoroughly addressed.

In our recent paper [19], the main focus was on the morphology of nanoparticles generated in the GWG. An anomalous nanoparticle size evolution was demonstrated in the modified GWG configuration, in which two parallel wires were employed. It was speculated that the found peculiarities originate from a competition between the particle surface growth and nucleation. The latter appeared to be a non-independent process on each wire that was affected by the proximity of the other wire. Although our speculations seem reasonable, the experimental approach in Fomenko et al. [19] does not allow for a direct isolation of the phenomenon related to the nucleation rate in the system.

In the current paper, we are focusing on the GWG’s nucleation performance in the same wire configurations varying only the orientation of the glowing wire with respect to the gas flow. Such arrangements enable altering paths of nucleating aerosols over particulate-generating wires, depending on the geometry to be used. In particular, an effective distance between the neighbouring wires is changed and the corresponding investigation on the mutual influence of the wires on the nanoparticle generation could be undertaken.

## 2. Materials and Methods

**Figure 1:**
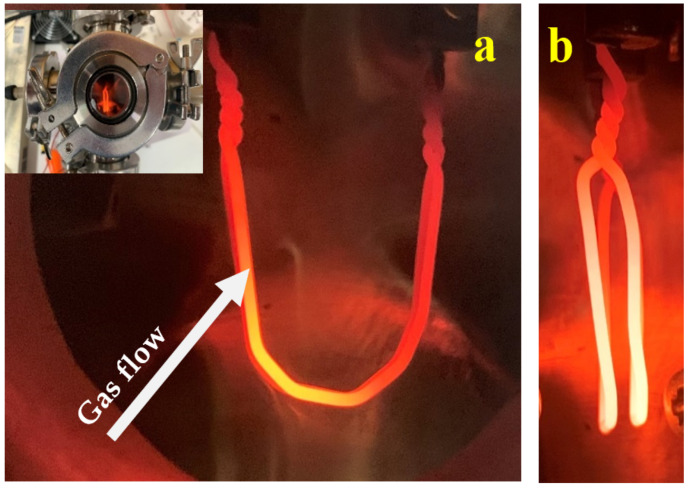
Photo images of the double molybdenum wire glowing chamber. The gas flow is perpendicular to the plane: (**a**)—the gas flows through the “gate” formed by the wire (TH); (**b**)—the gas flows along the “gate” formed by the wire (AL). Inset illustrates GWG arrangement.

Molybdenum oxide nanoparticles were produced using a glowing wire generator (GWG, Model 3709, AeroNanoTech, Moscow, Russia) as described by Boskovic and Agranovski [20]. The experimental setup is shown in Figure 1, which consists of a set of molybdenum double wires (99.5%, 1 mm diameter) attached to two electrodes (distance between wires was approximately 3 mm, and the distance between electrodes was about 22 mm). The wires were heated with the electric current (applied voltage ranged from 0.90 to 1.20 V was controlled by EMS 10–100 power supply (Electronic Measurements Inc., Neptune, NJ, USA)) to produce aerosols. Two different ways of the wire orientation relative to the gas flow were used in the experiment. First (Figure 1a), the gas flow was made to pass perpendicularly through (TH) the “gate” formed by the wire, and second, (Figure 1b), the gas was arranged to flow along (AL) the wire “gate” plane. Such arrangements were achieved by turning the electrical terminal holder plate by 90 degrees. Two different flowrates of the carrier gas (>99.9% purity nitrogen, BOC Ltd., North Ryde NSW Australia) were selected, namely, 0.3 and 1 slm; based on our previous experience [19], those were expected to provide the most pronounced effect. The particulate output was characterized by a Scanning Mobility Particle Sizer (SMPS, Model 3080 Classifier along with 3775 CPC, TSI Inc., Shoreview, MN, USA). The particle size distribution (PSD) obtained by the SMPS was further integrated in order to infer the total particle concentration at the GWG exit.

## 3. Results and Discussion

**Figure 2:**
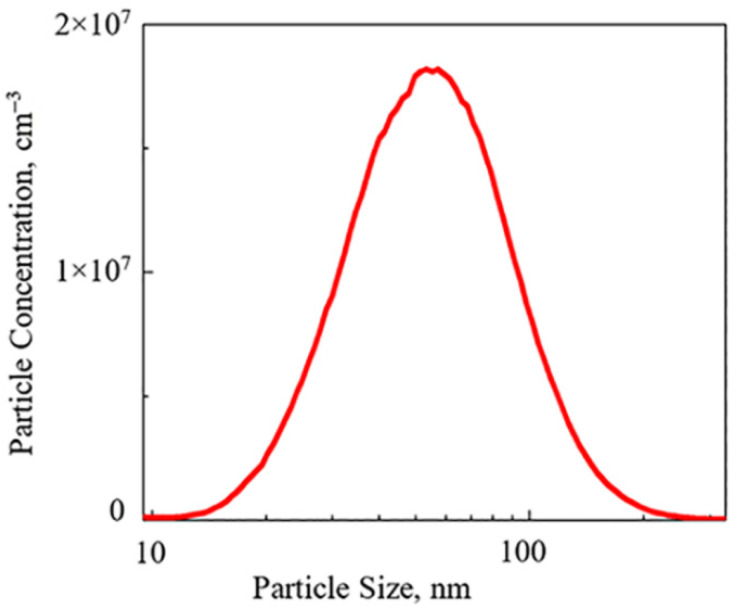
Typical PSD at voltage of 1 V, and flow rate of 1.05 slm obtained in TH configuration.

The typical PSD, which was further used to obtain the particle concentration, is shown in Figure 2. The area under the PSD, which is the concentration of particles generated in the GWG, is a measure of the nucleation rate in the system. It was calculated using the built-in Origin procedure at all studied conditions (applied voltage, gas flow rate) for both configurations.

**Figure 3:**
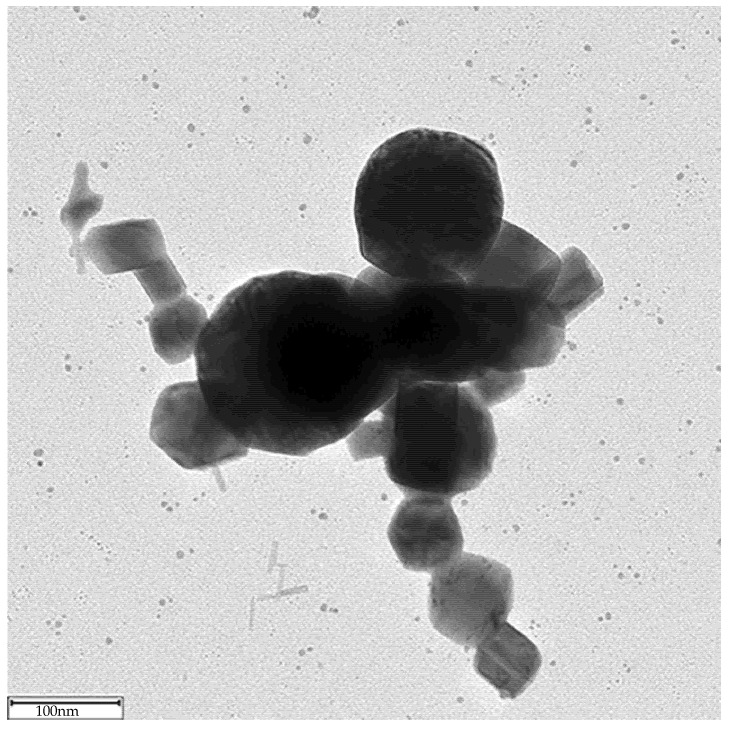
Typical morphology of molybdenum oxide nanoparticle obtained in AL configuration (adapted from [19]).

Some TEM images have been obtained to illustrate the particle morphology produced in the GWG. Figure 3 shows typical shape of molybdenum oxide nanoparticle as reported in [19].

In order to characterize a difference in nucleation under different geometries, two functions can be considered:

(1) $$F_{i}\equiv \frac{N_{TH}-N_{AL}}{N_{TH}}$$

and

(2) $$F_{d}\equiv \frac{2N_{AL}-N_{TH}}{N_{TH}}\;$$

where *N* is the particle concentration and subscripts “*TH*” and “*AL*” refer to the corresponding orientations. Based on the function definitions by Equations (1) and (2), one can see 2*·F_i_ + F_d_* = 1.

**Figure 4:**
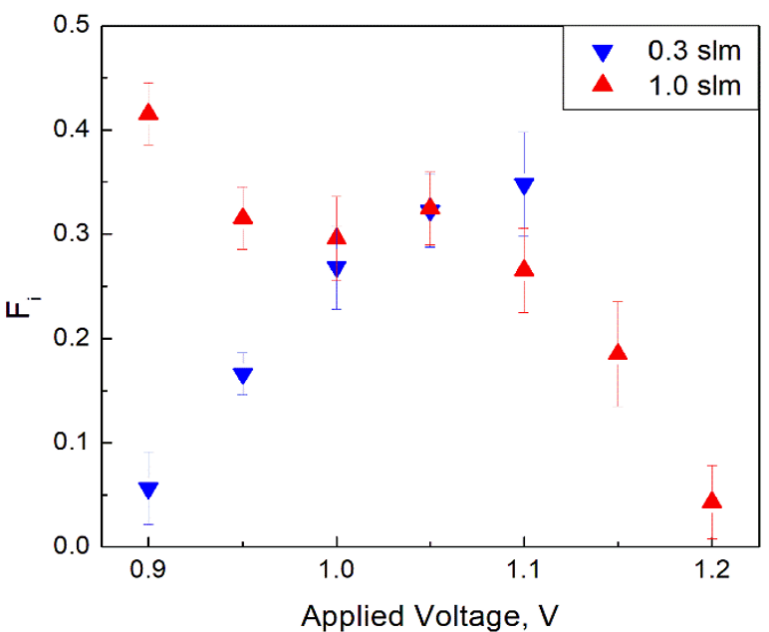
*F_i_* as a function of the applied voltage for two studied flow rates (Error bars represent STDEV of at least 20 measurements).

**Figure 5:**
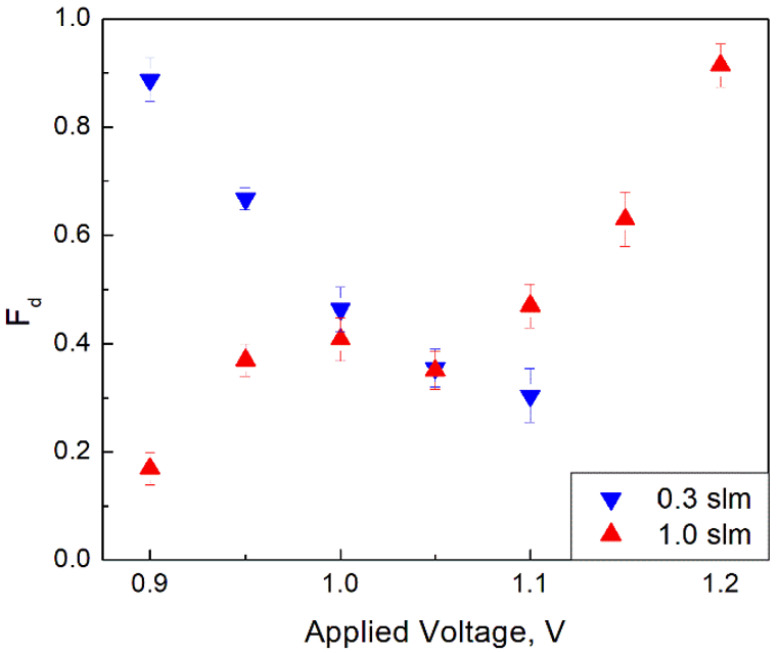
*F_d_* (see text for the definition) as a function of the applied voltage for two studied flow rates (Error bars represent STDEV of at least 20 measurements).

Figure 4 and Figure 5 show functions *F_i_* and *F_d_* at different applied voltages for two studied gas flowrates. Each data point corresponds to an average of 20 measurements taken at the given experimental parameters (applied voltage and gas flow). Error bars represent the experimental scatter (STDEV) of measured values.

In order to understand the physical meaning of the introduced functions *F_i_* and *F_d_* (see Equations (1) and (2)), the processes occurring on wires at different orientations with respect to the gas flow should be considered. Following Fomenko et al. [19], it is understandable that in the AL configuration, the particles formed on the wire part that sees the flow earlier can affect the nucleation of aerosols formed on the wire part that sees the flow later. This nucleation suppression occurs due to a preferential surface growth of already existing particles, which depletes the vapor concentration. In addition, the gas pre-heating also affects the process. Based on this notion, one can understand that if the effect is negligible, nucleation on the second part of the wire in the AL configuration would occur as the first part were non-existent. This could be called an “independent” aerosol formation. In that case, there would be no difference in nucleation rates between the TH and AL configurations, so *F_i_* = 0 and *F_d_* = 1. On the contrary, if the part of the wire in the AL configuration that sees the flow later does not produce additional particles, i.e., nucleation on the second part of the wire is completely suppressed (becomes “dependent”) and only particulate nucleated on the wire part, which sees the flow earlier, is detected, *F_d_* = 0 and *F_i_* = 0.5.

The above understanding is a guide to analyze results presented in Figure 4 and Figure 5. The aerosol formation is “dependent” either at the high voltage and low flow rate or at the low voltage and high flow rate. On the contrary, either at the low voltage and low flow rate or at the high voltage and high flow rate, the aerosol formation is rather independent.

As it was hypothesized by Fomenko et al. [19], the mechanism of the observed “dependency” of nucleation in the AL configuration is related to temperature coupling between two parts of the wire. The results of the current study support that idea. Indeed, the “dependency” becomes more significant in two cases: (1) when the voltage increases at the low flowrate; (2) when the flowrate increases at the low voltage. In both cases, more heat does transfer from one part of the wire to another.

## 4. Concluding Remarks

In the current study, we have directly demonstrated that particulate formation in the modified (double-wire) configuration of a GWG has important peculiarities related to the regime of nanoparticle nucleation within the device. The peculiarities appear as a dependence of the nucleation regime on the relative configurations of wires vs. the gas flow. Two extreme cases are possible, namely, (1) independent nucleation of nanoparticles on both parts of the wire that is achieved in the TH configuration, and (2) dependent nucleation of nanoparticles that is achieved at a certain set of parameters (applied voltage and gas flow) in the AL configuration. The latter should be taken into account when designing the multi-wire GWGs intended to enlarge produced nanoparticles, and further extend the device range for industrial applications. Additionally, the phenomenon found on the variation of the nanoparticle generation rate depending on the nucleating source orientation in the gas flow may have general implications for the mechanisms of nanoparticle formation [21] in flames during combustion.

## Data Availability

The data presented in this study are available on request from the corresponding author.
